# About the Action of Arsenious Acid upon the Tooth-Pulp

**Published:** 1897-03

**Authors:** H. Prinz

**Affiliations:** St. Louis, Mo.


### THE DENTAL REGISTER.

VOL. LI.] **MARCH, 1897.** [No. 3.

### COMMUNICATIONS.

### About the Action of Arsenious Acid upon the Tooth-Pulp

BY H. PRINZ, D.D.S., ST. LOUIS, MO.

There is probably no remedial agent in the whole dental materia medica, which is so universally used in the process of devitalization of the tooth-pulp as arsenious acid. Dr. Spooner, of the *Dominion Kingdom*, in 1836, called first attention to this particular effect and published a formulæ which has stood the test of more than half a century, and is used to-day, either as the original compound, or in various modifications.

Occasionally corrections have been suggested so as to render the application of the remedy to some extent painless, morphia-salts being preferred by many. The narcotic value of the drug in such compounds is rather doubtful; cocain as a local anæsthetic has a decided advantage over the opium alkaloid. Wood charcoal, as a diluent, and cobalt (cobalt arsenatis), as a substitute, have been highly praised at different periods by their advocates. The fact remains, nevertheless, unaltered, the arsenious acid is the only potent factor.

The mode and form of application varies with various practitioners. A paste made of the acid by the aid of some liquefying agent, such as creosote, phenol or etherial oils is almost universally used now. The action of the acid upon the pulp is twofold: first, as a caustic, and secondly, as a anæsthetic. But how this effect is produced has been rather questionable, and is still the cause of frequent discussions, and only shows the vital importance of the subject. Arkoevy makes the statement that, "after the application of the arsenious acid upon the pulp the blood-vessels dilate, the contents therein disintegrate, the connectives tissue-fibers become extended and the myelin of the nerve-fiber break up in nodules, while the axis-cylinders disappear entirely. The structure of the dentin remains unaltered." Baume and Schlenker do not confirm the last assertion.

Another hypothesis brought forward demonstrates the formation of a hyperæmia produced by the arsenious acid with resulting strangulation of the nerve-fibers near the apical foramen. This is not correct. We may, with the same effect, cauterize the pulps in young molars where the roots are not nearly fully developed. It should not be practiced, however. The arsenic may, by passing through the foramen, involve the periosteum and surrounding tissues, resulting in necrosis of the affected portion.

To ascertain the pharmaco-dynamic effect of arsenious acid some fundamental facts have been brought forward by the investigation of Bing and Schuly. They claim that the protoplasm in some forms of dead and living tissues have the power to interchange constantly the atoms of oxygen which adhere to the arsenic, viz.: to convert the arsenious acid ($As_2O_3$) in arsenic acid ($As_2O_4$) and *vice versa.* This permanent interchange of both the acids, this perpetual oxidation and reduction within the molecules of albumen causes a violent to and fro vibration of the atoms of oxygen, and this is the cause of the poisonous and the therapeutical effect. The metalloid arsenic acts merely as the carrying factor of the active molecules of oxygen which resembles somewhat the caustic properties of nitrogen dioxide ($N_2O_2$) and nitrogen terroxide ($N_2O_3$), the nitrogen itself being here the transporting medium.

Fibehue regards the members of the arsenic-phosphorus-antimony group not as remedial agents proper. Only such tissue as is directly diseased, or such as carries the pathological germ within will die, viz.: by the action of arsenic. On the healthy mucous membrane (in animal experiments), arsenic has no effect—in the animal, however, poisoned with arsenic, where the cells therefore have lost there resistance the arsenic will cauterize the mucous lining.

From exeriments carried on in the laboratories of the dental college in Vienna (Prof. Scheff), we learn that if the arsenical paste is brought in direct contact with the spread out mesentery of a frog, a momentary stasis of the circulation will result.

Microscopical pictures show most of the capillaries over-filled with clotted blood, few were found empty. The nodules in the vessel wall seemed to be somewhat extended. Healthy pulps of freshly-extracted teeth were subjected to the same treatment. After preparing them for histological researches they showed, in most cases, a closer coherence of the nodules in the capillaries and the vessels themselves were seemingly thinner and presented a wave-like appearance.

Diseased pulps were also subjected to the influence of the arsenical paste for twenty-four hours continuously. In longitudinal sections the specimens represented a picture somewhat different from the normal pulps. The capillaries were almost constantly filled with blood. The corpuscles, being brought in dense contact, looked similar to fibrin-coagulum. In some of the peripheral vessels the clot represented a conglomerate of disintegrated blood-disks and fibrin masses. The excessive accumulation had extend the lumen of the vessels abnormally. In a pathological pulp there is always inflammation and as an accompanying phenomenon hyperæmia is present. From the foregoing experiments the conclusion may be drawn; *that the complete stasis of the circulation is the immediate result of the action of the arsenical paste. In consequence of the stasis in the capillaries of the pulps the nerve-endings, and probably the nerve-branches lose their specific physiological action and in this way produce the analgesic effect.*

Heronan, in his text-book on physiology, explains this in the following manner:

In a total different relation to the circulation as the nerve itself, remains the central organs of the nerves, and the peripheral end-organs; stasis, viz.: the prolonged interruption of arteria circulation produces a much quicker disturbance of functions than in the muscles.

After the application of the arsenical paste the whole pulp will probably not break down; the newly-formed scab does not permit of deeper penetration of the caustic. Intoxication, perhaps, is excluded, the rapidly-formed scab prevents further involving of the whole pulps and this counts for the still painful sensation which our patients experience by the extirpation of the minute stumps from the root-canals. The quantity of arsenious acid applied to a tooth-pulp varies to some extent, we may safely say one-twentieth of a grain is the maximum amount in each individual case. More than two teeth should not be subjected to treatment at one sitting to prevent any possible chance of accidentally swallowing a large amount of the drug. Resulting failures find their explanation either in faulty application or it may be due to idiosyncracy.

